# A systematic review of sodium-glucose cotransporter 2 inhibitors and renal profiles among Japanese patients with type 2 diabetes mellitus

**DOI:** 10.1186/s40780-023-00305-x

**Published:** 2023-09-15

**Authors:** Junichi Mukai, Nakaba Okamura, Yuki Saito, Rie Kubota

**Affiliations:** Division of Clinical Pharmacy (Laboratory of Clinical Pharmacy Education) and Research and Education Center for Clinical Pharmacy, School of Pharmacy, Kitasato University, 5-9-1, Shirokane, Minato-ku, Tokyo, 108-8641 Japan

**Keywords:** Meta-analysis, Systematic review, Sodium-glucose co-transporter 2 inhibitors, Renal profiles, Type 2 diabetes mellitus, Japanese

## Abstract

**Background:**

We conducted a systematic review and meta-analysis to summarize the available literature and comprehensively appraise the renal profiles of sodium-glucose cotransporter 2 inhibitors (SGLT2i) in Japanese patients with type 2 diabetes mellitus (T2DM).

**Methods:**

The electronic databases MEDLINE, Ichushi-web, and ClinicalTrials.gov were searched for studies without language restrictions from their inception until 20 July 2023 and CENTRAL until 21 September 2021. Studies were included if they were double-masked randomized controlled trials (RCTs) (1) including Japanese patients with T2DM aged > 18 years who received SGLT2i or a placebo, (2) reporting at least one renal outcome of serum creatinine or the estimated glomerular filtration rate (eGFR), and (3) with a follow-up of > 12 weeks. Cross-over and open label trials were excluded. The risk of bias based on the Cochrane risk-of-bias tool for randomized trials (RoB 2) was appraised. We computed the weighed mean difference with 95%CI for each renal outcome and used a random-effects model (inverse variance method).

**Results:**

We ultimately retrieved 13 RCTs including 2687 individuals in our review. The durations of RCTs ranged between 12 and 104 weeks. Only one trial had a longer duration of more than one year. Ten out of 13 RCTs reported serum creatinine, while nine included eGFR. Serum creatinine and eGFR were slightly worse with SGLT2i than with a placebo [mean difference 0.01 (95%CI 0.00 to 0.02) mg/dL, p = 0.002, mean difference − 1.30 (95%CI -2.23 to -0.37) mL/min/1.73 m^2^, p = 0.006, respectively]. Merged results revealed insignificant heterogeneity (I^2^ < 30%).

**Conclusion:**

These results suggest that SGLT2i slightly worsens serum creatinine and eGFR in Japanese patients with T2DM. However, since the durations of most RCTs were short, the effects of eGFR in particular may be transient. Further evidence is needed from rigorous studies that focus on renal outcomes with a longer duration to confirm the present results.

**Supplementary Information:**

The online version contains supplementary material available at 10.1186/s40780-023-00305-x.

## Background

Sodium-glucose cotransporter 2 inhibitors (SGLT2i) were developed as a glucose-lowering medication that inhibits glucose reabsorption in proximal tubules and increases urinary glucose excretion. Large trials demonstrated that SGLT2i, such as empagliflozin (EMPA), canagliflozin (CANA), and dapagliflozin (DAPA), exerted reno-protective effects in patients with type 2 diabetes mellitus (T2DM) [1–3].

In contrast, meta-analyses of patients with T2DM showed no significant differences in the estimated glomerular filtration rate (eGFR) between SGLT2i and a control or placebo [4–6]. However, these studies were consistently affected by statistical heterogeneity and had a broad population in terms of participants. Furthermore, the large trials described above had racial diversity, including > 50% Caucasians [1–3]. Additionally, a recent meta-analysis of 14 randomized controlled trials (RCTs) including Asian populations with T2DM showed that SGLT2i reduced eGFR and serum creatinine (SCr) [7]. One cohort study on SGLT2i users demonstrated that Black race was associated with an increased risk of eGFR dipping [8]. Based on these findings, it currently remains unclear whether renal profiles in patients with T2DM receiving SGLT2i are dependent on racial differences. Moreover, to the best of our knowledge, meta-analyses have not yet examined the renal effects of SGLT2i in a population restricted to Japanese patients with T2DM. Therefore, the aims of our systematic review and meta-analysis were to summarize the available literature and comprehensively appraise the renal profiles of SGLT2i in Japanese patients with T2DM.

## Methods

The protocol was not prepared. We followed the PRISMA 2020 statement and submitted check lists [9] (Additional file 1).

### Search strategies

We searched MEDLINE, Ichushi-web, and ClinicalTrial.gov from their inception to 20 July 2023 and Cochrane Central Register of Controlled Trials (CENTRAL) until 21 September 2021. We also collected information on six SGLT2i that have been approved in Japan: CANA, DAPA, EMPA, ipragliflozin (IPRA), luseogliflozin (LUSEO), and tofogliflozin (TOFO). Individual SGLT2i names, alternative names, and “SGLT2 inhibitors” were used as search terms (Additional file 2). Our search of these databases was restricted to “randomized controlled trials”. We did not use a language filter. We also used the advanced search mode of ClinicalTrial.gov in terms of an age older than 18 years using each SGLT2i name, type 2 diabetes, and Japan as the key words. At least two assessors (YS and JM or NO and JM in a team) independently undertook the literature search. Any discrepancies were settled through discussions between assessors. A reference search was also performed to identify more RCTs based on the studies retrieved where appropriate.

### Data collection

Studies were included if they were double-masked RCTs (1) including Japanese patients with T2DM older than 18 years who received SGLT2i or a placebo, (2) reporting at least one renal outcome of our interest: SCr, eGFR, and the percentage of subjects showing a decline in eGFR from baseline as renal dysfunction [10], and (3) with a follow-up of > 12 weeks. Cross-over studies, open-label studies, RCTs without information on race/ethnicity, RCTs involving healthy participants, and trial registries with no results posted were excluded from our review. Data were also collected on concomitant medication, subtypes of SGLT2i, daily doses of SGLT2i, the number of participants, the dropout rate during the double-blind period, trial durations for the double-blind period, age, HbA1c, body mass index (BMI), eGFR at baseline, and the presence or absence of cardiac disease. We described insufficient information in RCTs as unclear. The first reviewer (JM) extracted data. The second reviewer (NO) then carefully rechecked the data.

### Endpoint

eGFR was the primary endpoint and SCr was the secondary endpoint.

### Assessment of the risk of bias

Two assessors (NO and JM) independently appraised the risk of bias based on the Revised Cochrane risk-of-bias tool for randomized trials (RoB 2) [11]. Any discrepancies were also resolved through discussions between assessors. Five domains for the risk of bias were as follows: bias arising from the randomization process, bias due to deviations from intended interventions, bias due to missing outcome data, bias in the measurement of the outcome, and bias in the selection of the reported result. Each domain was labeled as a “low risk of bias”, “some concerns”, or a “high risk of bias”.

### Data synthesis

**Fig. 1:**
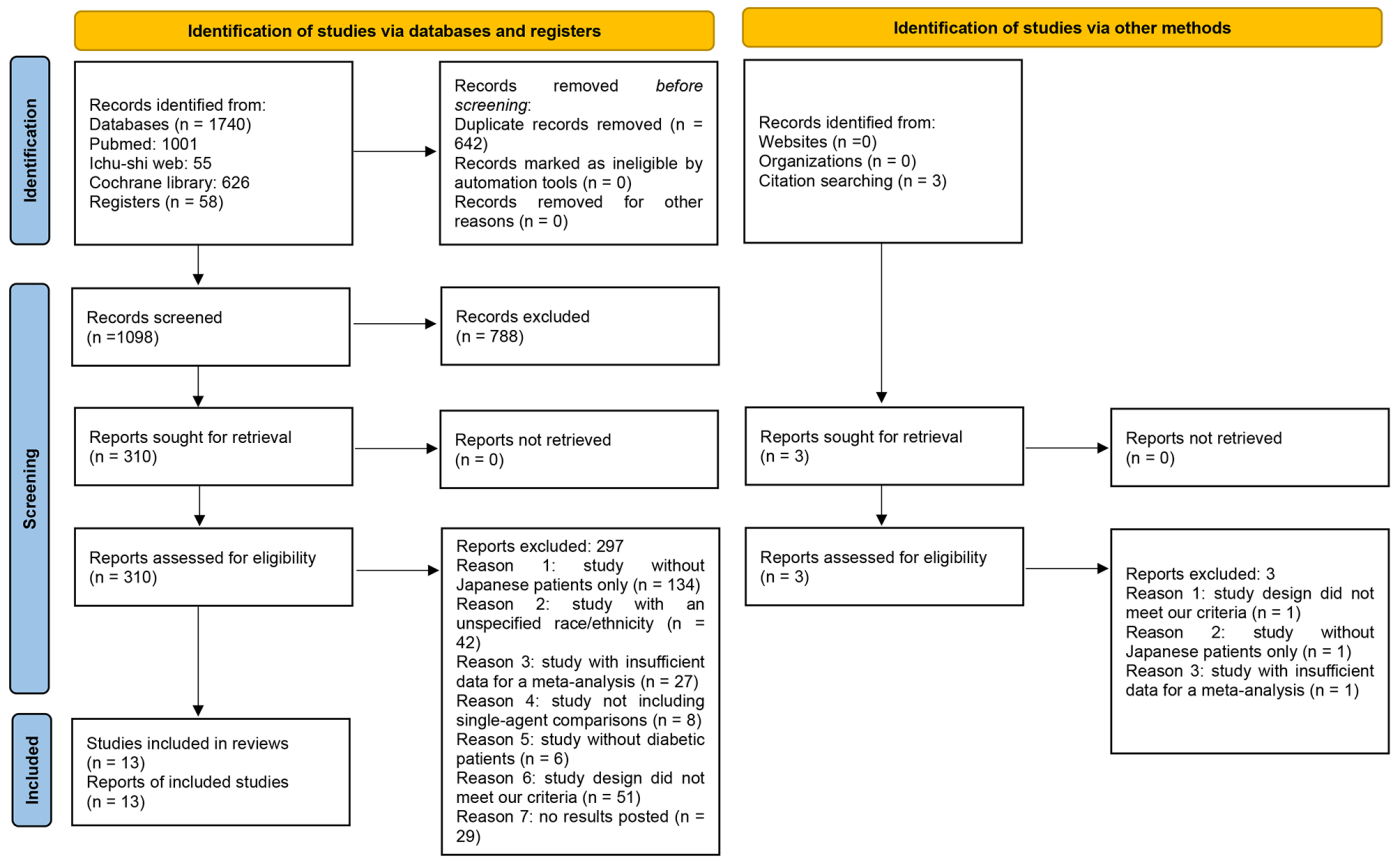
PRISMA2020 flow diagram

The first reviewer (JM) extracted changes from the baseline data of SCr and eGFR in both the intervention and placebo arms for the double-blind period using a spreadsheet; in instances where changes for the standard deviation were not reported directly (e.g., standard error of a mean or confidence interval (CI) for a mean), they were converted to the standard deviation for a mean using Review Manager 5.4.1 software (The Nordic Cochrane Centre, The Cochrane Collaboration, 2014). If the least squares mean was used in each RCT, we considered the value to be a mean. A RCT with insufficient data for analysis was excluded; for example, the number of subjects analyzed was unclear or the change from baseline data was not reported directly (Fig. 1). Multiple SGLT2i arms in a single RCT were combined into a single arm [12]. The second reviewer (NO) then carefully rechecked the data. In the meta-analysis, we computed the weighed mean difference (MD) with 95%CI for each renal outcome. The heterogeneity of each outcome was evaluated using chi-squared and I-squared statistics. A value of 40% or more represented marked heterogeneity [12]. We used a random-effects model (inverse variance method) to provide a conservative estimate. We depicted a forest plot for each outcome. Three post-hoc subgroup analyses were performed by including patients with T2DM and renal impairment only, with T2DM and normal renal function only, and those treated with SGLT2i as monotherapy only. We drew a funnel plot and used Egger’s test to assess the publication bias for each renal outcome. All statistical analyses were performed with Review Manager 5.4.1 software and Stata/MP 17.0 version (Stata Corp, College Station, TX, USA). A *P* value < 0.05 was considered to be significant. The certainty of a body of evidence was appraised using the Grading of Recommendations Assessment, Development and Evaluation (GRADE) approach. Evidence was rated as high, moderate, low, or very low.

## Results

Among 1740 studies in the database search, 252 full texts and 58 trials registries were retrieved after the removal of duplicates and the screening of titles and abstracts. We ultimately included 13 RCTs in our systematic review. Figure 1 shows the process used to identify eligible RCTs [10, 13–24] following a PRISMA 2020 flow diagram [9]. Table 1 shows the characteristics of RCTs included in the review. Four types of SGLT2i were collected: CANA, IPRA, LUSEO, and TOFO. DAPA and EMPA studies were not retrieved in our review process because they did not meet our criteria. The durations of RCTs ranged between 12 and 104 weeks. Among 13 RCTs, three included patients with T2DM and renal impairment [10, 15, 16]. All trials were published in English.

**Table 1:** Baseline characteristics in 13 randomized controlled trials

Study name	Concomitant medications	Doses [mg/day]	N^a^	Dropout rate^b^ %	Duration weeks	Age year	HbA1c %	BMI kg/m^2^	eGFR mL/min/1.73 m^2^	Cardiac disease N
Wada 2022 [10]	ARB or ACEI	CANA 100 mg	154	18.5	108	62.5	7.8	26.9	55.7	Unclear
		Placebo	154							
Kaku 2021 [13]	Sitagliptin	IPRA 50 mg	73	4.9	24	61.0	8.1	25.7	82.0	Unclear
		Placebo	70			60.0	8.0	26.0	83.4	
Seino 2018 [14]	Insulin	LUSEO 2.5 mg	159	5.6	16	57.4	8.7	25.4	86.5	Unclear
		Placebo	74			57.1	8.8	25.2	87.9	
Haneda 2016^c^ [15]	Unclear	LUSEO 2.5 mg	95	5.5	24	67.9	7.7	25.5	52.0	19
		Placebo	50			68.4	7.7	25.8	52.4	10
Kashiwagi 2015 A [16]	Antidiabetic agents	IPRA 50 mg	118	9.7	24	63.9	7.5	25.8	60.2	Unclear
		Placebo	46			65.7	7.6	25.0	62.7	
Kashiwagi 2015B [17]	Sulfonylurea	IPRA 50 mg	165	12.8	24	59.6	8.4	25.8	84.2	Unclear
		Placebo	75			59.8	8.3	24.2	85.9	
Kashiwagi 2015 C [18]	Pioglitazone	IPRA 50 mg	97	12.5	24	56.2	8.2	27.1	90.6	Unclear
		Placebo	54			56.1	8.4	27.1	91.6	
Kashiwagi 2015D [19]	None	IPRA 50 mg	62	10.8	16	60.6	8.4	25.3	87.8	Unclear
		Placebo	67			58.3	8.3	25.6	86.1	
Inagaki 2014 [20]	None	CANA 100 mg	90	11.4	24	58.4	8.0	25.6	81.4	Unclear
		CANA 200 mg	88			57.4	8.0	25.4	87.2	
		Placebo	93			58.2	8.0	25.9	84.7	
Kaku 2014 [21]	None	TOFO 10 mg	57	8.5	24	58.6	8.5	25.1	84.9	Unclear
		TOFO 20 mg	58			56.6	8.3	25.0	86.8	
		TOFO 40 mg	58			57.0	8.4	25.8	86.0	
		Placebo	56			56.8	8.4	26.0	83.8	
Seino 2014 A [22]	None	LUSEO 1 mg	55	2.8	12	58.5	7.8	24.5	NR	Unclear
		LUSEO 2.5 mg	56			57.4	8.1	24.8	NR	
		LUSEO 5 mg	54			57.3	7.9	26.4	NR	
		LUSEO 10 mg	58			59.6	8.0	23.4	NR	
		Placebo	57			57.1	7.9	25.2	NR	
Seino 2014B [23]	None	LUSEO 0.5 mg	60	2.9	12	55.2	8.2	25.4	NR	Unclear
		LUSEO 2.5 mg	61			58.3	8.1	24.8	NR	
		LUSEO 5 mg	61			56.8	8.2	24.5	NR	
		Placebo	54			57.6	7.9	25.2	NR	
Seino 2014 C [24]	None	LUSEO 2.5 mg	79	6.3	24	58.9	8.1	26.0	NR	Unclear
		Placebo	79			59.6	8.2	25.3	NR	

CANA, canagliflozin; IPRA, ipragliflozin; LUSEO, luseogliflozin; TOFO, tofogliflozin; N, number of participants; BMI, body mass index; eGFR, estimated glomerular filtration rate, NR, not reported. ARB, angiotensin II receptor blocker; ACEI, angiotensin-converting enzyme inhibitor, ^a^Number of participants included in a full analysis set, ^b^Dropout rate during the double-blind period, ^c^The authors did not state a full-analysis set.

### Quality assessment of each RCT

**Fig. 2:**
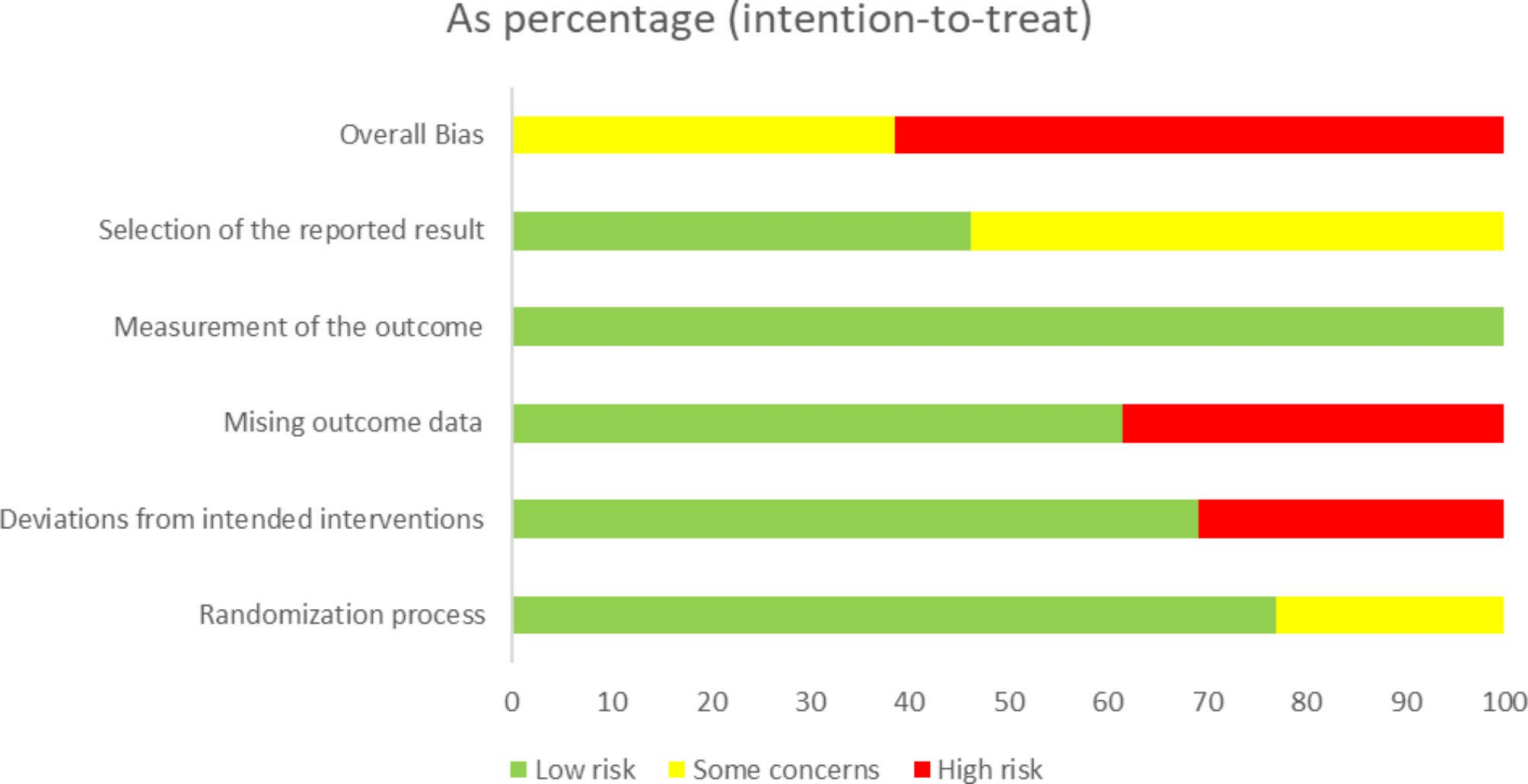
Risk of bias assessment using Version 2 of the Cochrane risk-of-bias tool for randomized trials

**Fig. 3:**
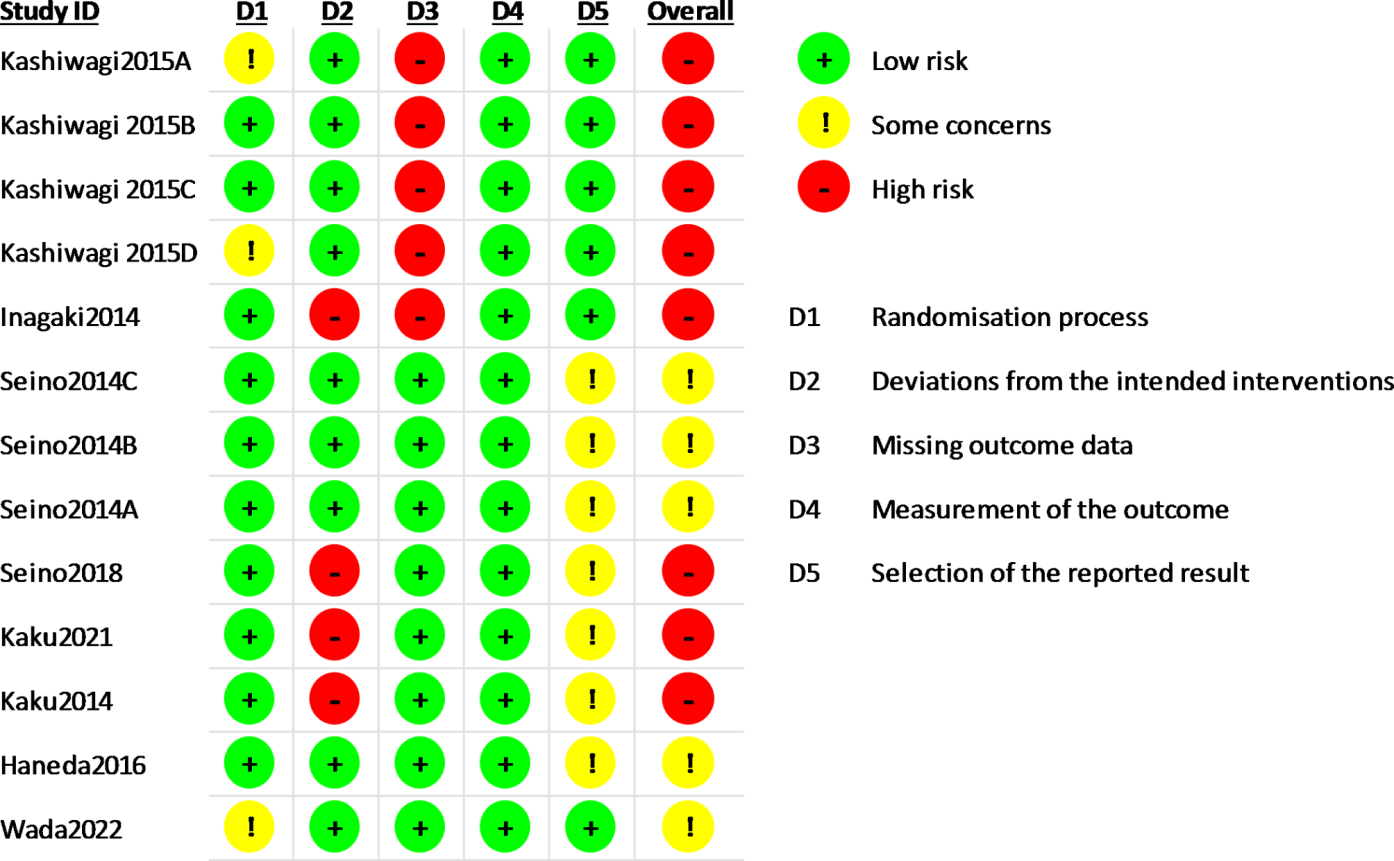
Risk of bias in individual studies

We assessed the risk of bias of RCTs based on RoB 2 [11]. The majority of studies were judged as having a “high risk of bias”. A “low risk of bias” was the highest in the domain of the measurement of the outcome. “Some concerns” was the highest in the domain of the selection of the reported result. A “high risk of bias” was the highest in the domains of missing outcome data (Figs. 2 and 3).

### SCr

**Fig. 4:**
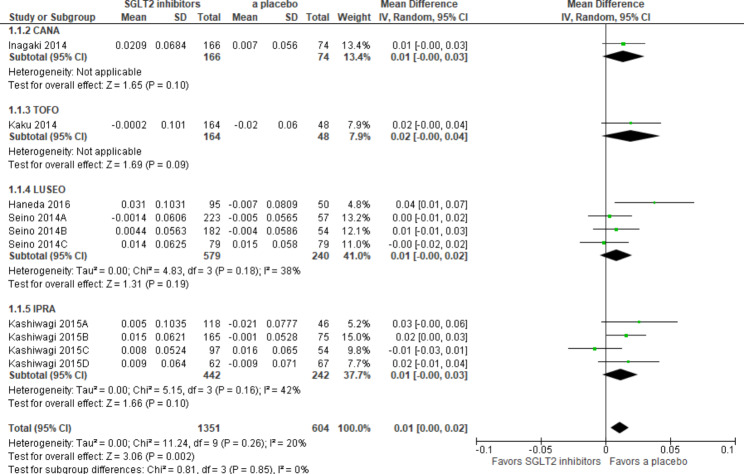
Forest plot for SCr. Abbreviations: SCr, serum creatinine; CANA, canagliflozin; IPRA, ipragliflozin; LUSEO, luseogliflozin; TOFO, tofogliflozin; SGLT2, sodium-glucose cotransporter 2

Among 13 studies, 10 RCTs were included in the meta-analysis. No heterogeneity was observed among RCTs (I^2^ = 20%). SCr values were slightly worse with SGLT2i than with a placebo [MD 0.01 (95%CI 0.00 to 0.02) mg/dL, *p* < *0.002*], whereas no significant differences were noted in a sub-group analysis of all subtypes of SGLT2i (Fig. 4).

### eGFR

**Fig. 5:**
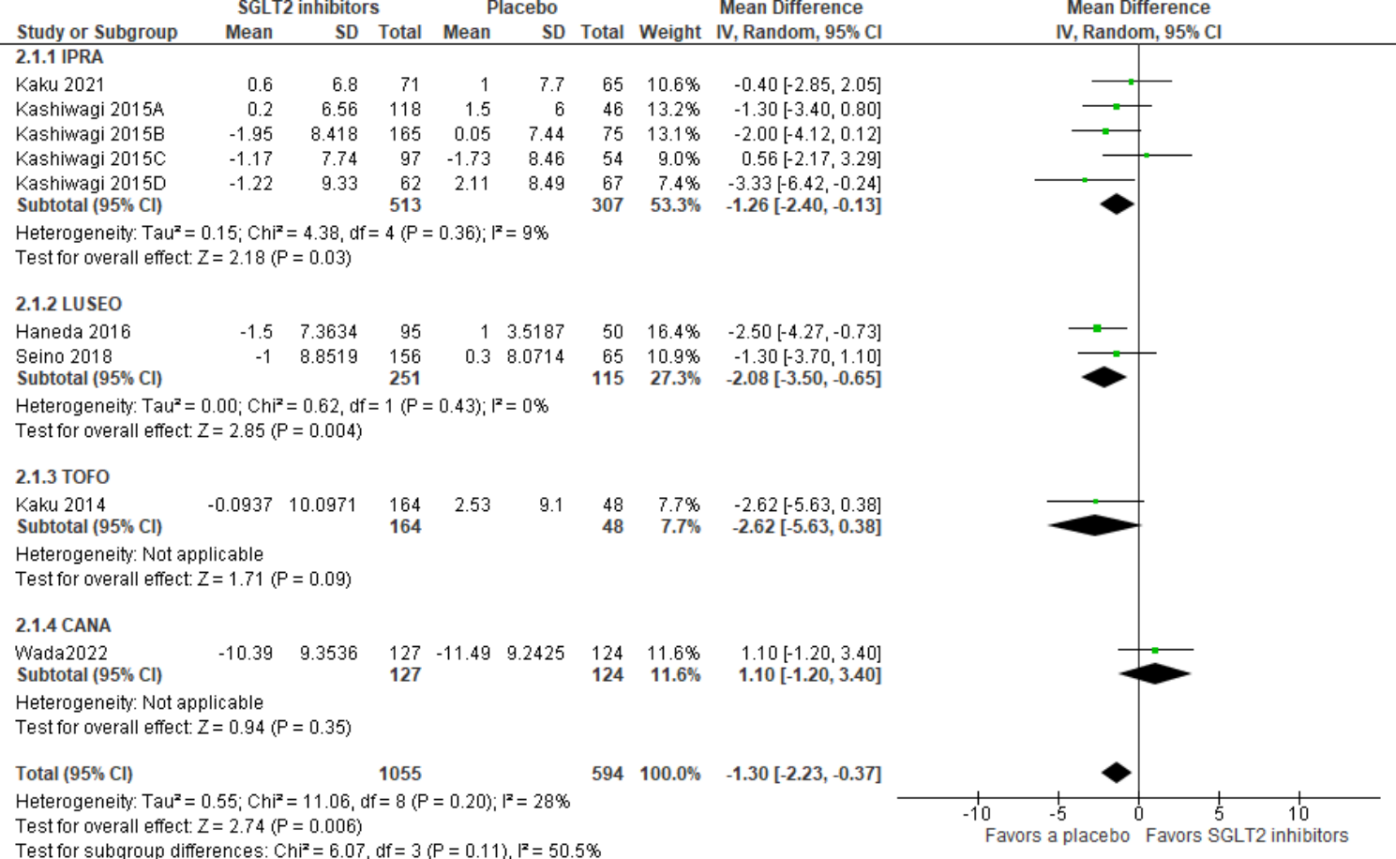
Forest plot for eGFR. Abbreviations: eGFR, estimated glomerular filtration rate; CANA, canagliflozin; IPRA, ipragliflozin; LUSEO, luseogliflozin; TOFO, tofogliflozin; SGLT2, sodium-glucose cotransporter 2

Among 13 studies, nine RCTs were included in the meta-analysis. No heterogeneity was observed among RCTs (I^2^ = 28%). eGFR values were slightly worse with SGLT2i than with a placebo [MD -1.30 (95%CI -2.23 to -0.37) mL/min/1.73 m^2^, *p* = 0.006], and TOFO and CANA were not significant in the sub-group analysis (Fig. 5).

### Incidence of a decline in eGFR from baseline

We were unable to combine data because only one study reported the incidence of a decline in eGFR [10].

### Publication bias

A publication bias was not observed for SCr or eGFR (Additional files 3–4).

### Additional analyses

The sub-group analysis of patients with T2DM and renal impairment only showed inconsistent main outcomes. The effect size of SCr among patients with T2DM and renal impairment only was greater than those with T2DM and normal renal function only. The effect size of eGFR among patients with T2DM and renal impairment only was smaller than those with T2DM and normal renal function only, but was not significant. The sub-group analysis including patients receiving SGLT2i monotherapy only was consistent with the main results (Table 2).

**Table 2:** Summary of subgroup analyses

	Outcome	Trial, n	Study name(s) merged	SGLT2i, n	Placebo, n	MD [95%CI]	I^2^, %	*p*
Only patients with T2DM and renal impairment	SCr, mg/dL	2	Haneda 2016 [15] Kashiwagi 2015 A [16]	213	96	0.03 [0.01, 0.05]	0	0.003
	eGFR, mL/min/1.73 m^2^	3	Haneda 2016 [15] Kashiwagi 2015 A [13] Wada 2022 [10]	340	220	-1.00 [-3.04, 1.03]	66	0.33
Only patients with T2DM and normal renal function	SCr, mg/dL	8	Inagaki 2014 [20] Kaku 2014 [21] Kashiwagi 2015B [17] Kashiwagi 2015 C [18] Kashiwagi 2015D [19] Seino 2014 A [22] Seino 2014B [23] Seino 2014 C [24]	1138	508	0.01 [0.00, 0.02]	0	0.008
	eGFR, mL/min/1.73 m^2^	6	Kaku 2014 [21] Kaku 2021 [13] Kashiwagi 2015B [17] Kashiwagi 2015 C [18] Kashiwagi 2015D [19] Seino 2018 [14]	715	374	-1.43 [-2.48, -0.37]	1	0.008
Only patients with T2DM treated with a SGLT2i as monotherapy	SCr, mg/dL	6	Inagaki 2014 [20] Kaku 2014 [21] Kashiwagi 2015D [19] Seino 2014 A [22] Seino 2014B [23] Seino 2014 C [24]	876	379	0.01 [0.00, 0.02]	0	0.02
	eGFR, mL/min/1.73 m^2^	2	Kaku 2014 [21] Kashiwagi 2015D [19]	226	115	-2.97 [-5.12, -0.81]	0	0.007

SGLT2i sodium-glucose cotransporter 2 inhibitors, T2DM type 2 diabetes mellitus, NA not applicable, I^2^ heterogeneity, SCr serum creatinine, eGFR estimated glomerular filtration rate, MD weighted mean difference.

### Evaluation for GRADE

We rated the certainty of evidence for SCr as low. We downgraded the SCr outcome because six studies had a high risk of bias in terms of missing outcome data or deviations from the intended interventions, and 95%CI was near to zero for imprecision. We rated the certainty of evidence for eGFR as moderate. We downgraded the eGFR outcome because seven studies had a high risk of bias in terms of missing outcome data or deviations from the intended interventions (Fig. 3, Additional file 5).

## Discussion

We conducted a systematic review and meta-analysis to summarize the available literature and comprehensively appraise the renal profiles of SGLT2i in Japanese patients with T2DM. eGFR and Scr were slightly worse with SGLT2i than with a placebo. Merged results revealed insignificant heterogeneity (I^2^ < 30%).

Our analysis showed that SCr was significantly higher with SGLT2i than with a placebo; the results obtained on the subtypes of SGLT2i were not significant, whereas merged results on subtypes of SGLT2i were significant [MD 0.01 (95%CI 0.00 to 0.02) mg/dL, *p* < *0.002*] (Fig. 4). The LUSEO or IPRA group with a larger sample size may have caused this result in the forest plot. However, this undesirable effect of SGLT2i may be negligible. A previous meta-analysis that included more than 50% of Japanese patients with T2DM without renal impairment who received SGLT2i showed no significant differences in SCr between SGLT2i and a placebo [5]. Furthermore, Inagaki et al. reported that an increase in SCr due to SGLT2i did not explain the attenuation of renal function because the change was not progressive and UACR did not increase with higher SCr [20]. Two previous meta-analyses of T2DM showed that UACR was consistently lower with SGLT2i than with a placebo [4, 6].

The present results revealed a significantly larger reduction in eGFR in the SGLT2i group than in the placebo group; however, our trial duration ranged between 12 and 104 weeks. SGLT2i has been shown to transiently decrease eGFR within the initial four weeks and thereafter return it to the baseline level, which is often referred to as the eGFR dip [1, 3, 25]. A previous meta-analysis that merged two large RCTs including patients with T2DM and chronic kidney disease showed that the decline in the eGFR slope (rate of change in eGFR from week four to the last measurement within a double-blind period) was slower with SGLT2i than with a placebo [2, 26, 27]. However, we were unable to combine eGFR at the initial dip because only one RCT conducted by Kashiwagi et al. presented both the mean and standard deviation of eGFR at the initial dip; the value of eGFR at week two in their study was the lowest at any timepoint measured throughout the treatment period [16]. Besides the short duration of the RCTs we observed, the different assessment time points of eGFR may explain the opposite result of a large RCT showing that SGLT2i administered for one year or longer to patients with T2DM slowed the reduction in eGFR more than a placebo: one large-scale RCT showed that eGFR at baseline in the SGLT2i arm declined within the first four weeks and then gradually recovered after four weeks, while the level of eGFR on SGLT2i was roughly matched with that of the placebo arm at 52 weeks [1]. Similarly, in one matched cohort study using a Japanese database that included patients with T2DM and with a median observation period of 17 months, the reduction in eGFR was slower with SGLT2i than with other glucose-lowering medications [28]. Therefore, more domestic RCTs with longer durations that evaluate eGFR over time are needed to verify the different findings obtained from Japanese populations. One large-scale longitudinal study reported that the rate of the annual decline in eGFR among a general Japanese population was 0.36 mL/min/1.73 m^2^/year [29]. The impact of eGFR in the present analysis may have been transient and the annual decline in eGFR may also partially account for the results obtained. A pooled analysis consisting of four phase 3 studies that included Japanese individuals with a mean HbA1c of 7.8% also showed that an acute decline in eGFR after the initiation of SGLT2i was inversely associated with age, BMI, and eGFR at baseline [30]. Furthermore, a similar result for eGFR was observed in chronic kidney disease patients without diabetes [31]. Therefore, an acute change in eGFR among patients with these characteristics needs to be closely monitored in the early stage after initiating SGLT2i, and recovery from the eGFR dip needs to be confirmed.

Our sub-analysis suggested that the impact of SGLT2i on eGFR was weak among Japanese patients with T2DM and renal impairment [MD -1.00 (95%CI -3.04 to 1.03) mL/min/1.73 m^2^]. Consistently, this parameter may return to baseline values over time even in the presence of CKD [3, 32]. Furthermore, when the RCT with a longer follow-up period of 104 weeks was excluded [10], our results on the overall population revealed a more negative impact on eGFR: the eGFR value was worse with SGLT2i than with a placebo [MD -1.64 (95%CI -2.47 to -0.81) mL/min/1.73 m^2^]. The CANVAS study, which included approximately 80% Caucasians, showed that eGFR gradually recovered over several years irrespective of mean eGFR at baseline [33]. Additionally, the percentage of eGFR non-dippers receiving SGLT2i was similar between a Japanese population and EMPA-REG OUTCOME, including approximately 70% Caucasians; however, BMI was lower in the Japanese population than in the Caucasians [25, 30]. Therefore, race/ethnicity may not have an impact on changes in eGFR in a sufficient time window.

The present study has a number of strengths. To the best of our knowledge, this is the first systematic literature review and meta-analysis to summarize the available literature and comprehensively appraise the renal profiles of SGLT2i in Japanese patients with T2DM. Additionally, our merged outcomes consistently had acceptable heterogeneity (I^2^ < 30%). However, the present study also had several limitations. Therefore, the present results need to be interpreted with caution. There was a likelihood of a publication bias because we mostly identified published data. Another limitation is that not all of the RCTs collected set the outcomes of our interest as the primary outcome, and the majority of RCTs were judged as a “high risk of bias”, as described above. Furthermore, DAPA and EMPA, which exert reno-protective effects, were not included in the present analysis, which may have led to an under- or overestimation. Our study had limited information on dipeptidyl peptidase 4 inhibitors, which have the highest prescription rate as a first-line treatment for T2DM in Japan [34], or on renin–angiotensin system inhibitors, which exert reno-protective effects [35]. Moreover, an elderly population as one of the risk factors associated with chronic kidney disease was not included in our analysis [36]. This information is needed to verify the present results for their use in future studies. Another limitation was that neither SCr nor eGFR may have been accurate because we treated the least squares mean as the mean; however, we confirmed that similar results were obtained when these RCTs were excluded [10, 22–24]. Moreover, the present study may not truly reflect current clinical settings because the RCTs analyzed included highly selective populations that were rigorously controlled. Therefore, real-world evidence from diverse conditions is needed to confirm the present results.

## Conclusion

The present study, which included Japanese patients with T2DM only, suggests that SCr and eGFR were slightly worse with SGLT2i than with a placebo. There was also no important heterogeneity. However, since the durations of the RCTs included were mostly short, the effects of eGFR in particular may be transient. Further evidence is needed from rigorous studies that focus on renal outcomes for a longer duration and involve subtypes, such as DAPA and EMPA, to confirm the present results.

### Electronic supplementary material

Below is the link to the electronic supplementary material.

Supplementary Material 1

## Data Availability

The datasets used and/or analyzed during the present study are available from the corresponding author on reasonable request.

## Abbreviations

CANA: Canagliflozin
LUSEO: Luseogliflozin
IPRA: Ipragliflozin
TOFO: Tofogliflozin
SGLT2i: Sodium-glucose co-transporter 2 inhibitor
eGFR: Estimated glomerular filtration rate
SCr: Serum creatinine
MD: Weighted mean difference
T2DM: Type 2 diabetes mellitus
RCTs: Randomized controlled trials
BMI: Body mass index
RoB 2: The Revised Cochrane risk-of-bias tool for randomized trials
