# Supplementary material for: A systematic review of sodium-glucose cotransporter 2 inhibitors and renal profiles among Japanese patients with type 2 diabetes mellitus
Source: J Pharm Health Care Sci. 2023 Sep 15;9:36. doi: 10.1186/s40780-023-00305-x (PMC10504754; doi:10.1186/s40780-023-00305-x)
Supplement: Supplementary file 1 — Supplementary Material 1 [file 40780_2023_305_MOESM1_ESM.docx]

Supplementary information

A systematic review of sodium-glucose cotransporter 2 inhibitors and renal profiles in Japanese patients with type 2 diabetes mellitus

Junichi Mukai, Nakaba Okamura, Yuki Saito, and Rie Kubota

Additional file 1 PRISMA 2020 Checklist

# PRISMA 2020 Main Checklist

| **Topic** | **No.** | **Item** | **Location where item is reported** |
| --- | --- | --- | --- |
| **TITLE** |  |  |  |
| **Title** | 1 | Identify the report as a systematic review. | Title |
| **ABSTRACT** |  |  |  |
| **Abstract** | 2 | See the PRISMA 2020 for Abstracts checklist |  |
| **INTRODUCTION** |  |  |  |
| **Rationale** | 3 | Describe the rationale for the review in the context of existing knowledge. | Lines 43-58 |
| **Objectives** | 4 | Provide an explicit statement of the objective(s) or question(s) the review addresses. | Lines 58-60 |
| **METHODS** |  |  |  |
| **Eligibility criteria** | 5 | Specify the inclusion and exclusion criteria for the review and how studies were grouped for the syntheses. | Lines 74-77, 80-85, 104-113 |
| **Information sources** | 6 | Specify all databases, registers, websites, organisations, reference lists and other sources searched or consulted to identify studies. Specify the date when each source was last searched or consulted. | Lines 67-68, 76-77 |
| **Search strategy** | 7 | Present the full search strategies for all databases, registers and websites, including any filters and limits used. | Additional file 2, lines 71-74 |
| **Selection process** | 8 | Specify the methods used to decide whether a study met the inclusion criteria of the review, including how many reviewers screened each record and each report retrieved, whether they worked independently, and if applicable, details of automation tools used in the process. | Lines 74-76 |
| **Data collection process** | 9 | Specify the methods used to collect data from reports, including how many reviewers collected data from each report, whether they worked independently, any processes for obtaining or confirming data from study investigators, and if applicable, details of automation tools used in the process. | Lines 89-90, 104-108, 112-113. |
| **Data items** | 10a | List and define all outcomes for which data were sought. Specify whether all results that were compatible with each outcome domain in each study were sought (e.g. for all measures, time points, analyses), and if not, the methods used to decide which results to collect. | Line 93, lines 104-113 |
|  | 10b | List and define all other variables for which data were sought (e.g. participant and intervention characteristics, funding sources). Describe any assumptions made about any missing or unclear information. | Lines 85-89 |
| **Study risk of bias assessment** | 11 | Specify the methods used to assess risk of bias in the included studies, including details of the tool(s) used, how many reviewers assessed each study and whether they worked independently, and if applicable, details of automation tools used in the process. | Lines 96-101 |
| **Effect measures** | 12 | Specify for each outcome the effect measure(s) (e.g. risk ratio, mean difference) used in the synthesis or presentation of results. | Lines 113-114 |
| **Synthesis methods** | 13a | Describe the processes used to decide which studies were eligible for each synthesis (e.g. tabulating the study intervention characteristics and comparing against the planned groups for each synthesis (item 5)). | Lines 104-113 |
|  | 13b | Describe any methods required to prepare the data for presentation or synthesis, such as handling of missing summary statistics, or data conversions. | Lines 104-113 |
|  | 13c | Describe any methods used to tabulate or visually display results of individual studies and syntheses. | Lines 116-117 |
|  | 13d | Describe any methods used to synthesize results and provide a rationale for the choice(s). If meta-analysis was performed, describe the model(s), method(s) to identify the presence and extent of statistical heterogeneity, and software package(s) used. | Lines 113-117 |
|  | 13e | Describe any methods used to explore possible causes of heterogeneity among study results (e.g. subgroup analysis, meta-regression). | Lines 117-119 |
|  | 13f | Describe any sensitivity analyses conducted to assess robustness of the synthesized results. | NA |
| **Reporting bias assessment** | 14 | Describe any methods used to assess risk of bias due to missing results in a synthesis (arising from reporting biases). | Lines 119-120 |
| **Certainty assessment** | 15 | Describe any methods used to assess certainty (or confidence) in the body of evidence for an outcome. | Lines 122-124 |
| **RESULTS** |  |  |  |
| **Study selection** | 16a | Describe the results of the search and selection process, from the number of records identified in the search to the number of studies included in the review, ideally using a flow diagram. | Lines 128-131 Fig. 1 |
|  | 16b | Cite studies that might appear to meet the inclusion criteria, but which were excluded, and explain why they were excluded. | Lines 127-130, Fig. 1 |
| **Study characteristics** | 17 | Cite each included study and present its characteristics. | Lines 130-134, table 1 |
| **Risk of bias in studies** | 18 | Present assessments of risk of bias for each included study. | Lines 137-140 |
| **Results of individual studies** | 19 | For all outcomes, present, for each study: (a) summary statistics for each group (where appropriate) and (b) an effect estimate and its precision (e.g. confidence/credible interval), ideally using structured tables or plots. | Lines 143-156 |
| **Results of syntheses** | 20a | For each synthesis, briefly summarise the characteristics and risk of bias among contributing studies. | Lines 137-140, additional file 5 |
|  | 20b | Present results of all statistical syntheses conducted. If meta-analysis was done, present for each the summary estimate and its precision (e.g. confidence/credible interval) and measures of statistical heterogeneity. If comparing groups, describe the direction of the effect. | Lines 143-156 |
|  | 20c | Present results of all investigations of possible causes of heterogeneity among study results. | Lines 162-167 |
|  | 20d | Present results of all sensitivity analyses conducted to assess the robustness of the synthesized results. | NA |
| **Reporting biases** | 21 | Present assessments of risk of bias due to missing results (arising from reporting biases) for each synthesis assessed. | Line 159 |
| **Certainty of evidence** | 22 | Present assessments of certainty (or confidence) in the body of evidence for each outcome assessed. | Lines 170-175 |
| **DISCUSSION** |  |  |  |
| **Discussion** | 23a | Provide a general interpretation of the results in the context of other evidence. | Lines 182-221 |
|  | 23b | Discuss any limitations of the evidence included in the review. | Lines 243-248, 250-253 |
|  | 23c | Discuss any limitations of the review processes used. | Lines 239-243, lines 248-250 |
|  | 23d | Discuss implications of the results for practice, policy, and future research. | Lines 256-261 |
| **OTHER INFORMATION** |  |  |  |
| **Registration and protocol** | 24a | Provide registration information for the review, including register name and registration number, or state that the review was not registered. | Lines 63-64 |
|  | 24b | Indicate where the review protocol can be accessed, or state that a protocol was not prepared. | Lines 63-64 |
|  | 24c | Describe and explain any amendments to information provided at registration or in the protocol. | Lines 63-64 |
| **Support** | 25 | Describe sources of financial or non-financial support for the review, and the role of the funders or sponsors in the review. | Line 275 |
| **Competing interests** | 26 | Declare any competing interests of review authors. | Line 274 |
| **Availability of data, code and other materials** | 27 | Report which of the following are publicly available and where they can be found: template data collection forms; data extracted from included studies; data used for all analyses; analytic code; any other materials used in the review. | Lines 272-273 |

#####

# PRIMSA Abstract Checklist

| **Topic** | **No.** | **Item** | **Reported?** |
| --- | --- | --- | --- |
| **TITLE** |  |  |  |
| **Title** | 1 | Identify the report as a systematic review. | Yes |
| **BACKGROUND** |  |  |  |
| **Objectives** | 2 | Provide an explicit statement of the main objective(s) or question(s) the review addresses. | Yes |
| **METHODS** |  |  |  |
| **Eligibility criteria** | 3 | Specify the inclusion and exclusion criteria for the review. | Yes |
| **Information sources** | 4 | Specify the information sources (e.g. databases, registers) used to identify studies and the date when each was last searched. | Yes |
| **Risk of bias** | 5 | Specify the methods used to assess risk of bias in the included studies. | Yes |
| **Synthesis of results** | 6 | Specify the methods used to present and synthesize results. | Yes |
| **RESULTS** |  |  |  |
| **Included studies** | 7 | Give the total number of included studies and participants and summarise relevant characteristics of studies. | Yes |
| **Synthesis of results** | 8 | Present results for main outcomes, preferably indicating the number of included studies and participants for each. If meta-analysis was done, report the summary estimate and confidence/credible interval. If comparing groups, indicate the direction of the effect (i.e. which group is favoured). | Yes |
| **DISCUSSION** |  |  |  |
| **Limitations of evidence** | 9 | Provide a brief summary of the limitations of the evidence included in the review (e.g. study risk of bias, inconsistency and imprecision). | No |
| **Interpretation** | 10 | Provide a general interpretation of the results and important implications. | Yes |
| **OTHER** |  |  |  |
| **Funding** | 11 | Specify the primary source of funding for the review. | No |
| **Registration** | 12 | Provide the register name and registration number. | No |

*From:* Page MJ, McKenzie JE, Bossuyt PM, Boutron I, Hoffmann TC, Mulrow CD, et al. The PRISMA 2020 statement: an updated guideline for reporting systematic reviews. MetaArXiv. 2020, September 14. DOI: 10.31222/osf.io/v7gm2. For more information, visit: www.prisma-statement.org

Additional file 2 Search strategy for each database

Ichu-shi web:

((((Ipragliflozin/TH or イプラグリフロジン/AL)) or ((Dapagliflozin/TH or ダパグリフロジン/AL)) or ((Luseogliflozin/TH or ルセオグリフロジン/AL)) or ((Tofogliflozin/TH or トホグリフロジン/AL)) or ((Canagliflozin/TH or カナグリフロジン/AL)) or ((Empagliflozin/TH or エンパグリフロジン/AL)) or (("SGLT2 Inhibitors"/TH or SGLT2阻害薬/AL)))) and (RD=ランダム化比較試験)

PubMed:

Search ((((((("ipragliflozin" [Supplementary Concept])) OR ("dapagliflozin" [Supplementary Concept])) OR ("1,5-anhydro-1-(5-(4-ethoxybenzyl)-2-methoxy-4-methylphenyl)-1-thioglucitol" [Supplementary Concept])) OR ("6-((4-ethylphenyl)methyl)-3',4',5',6'-tetrahydro-6'-(hydroxymethyl)spiro(isobenzofuran-1(3H),2'-(2H)pyran)-3',4',5'-triol" [Supplementary Concept])) OR ("Canagliflozin"[Mesh])) OR ("empagliflozin" [Supplementary Concept])) OR ("Sodium-Glucose Transporter 2 Inhibitors"[Mesh]) Filters: Randomized Controlled Trial

Cochrane Central Register of Controlled Trials (CENTRAL):

1 ipragliflozin.mp.

2 dapagliflozin.mp.

3 luseogliflozin.mp. [mp=title, original title, abstract, mesh headings, heading words, keyword]

4 tofogliflozin.mp.

5 canagliflozin.mp.

6 empagliflozin.mp.

7 Sodium-Glucose Transporter 2 Inhibitors.mp.

8 1 or 2 or 3 or 4 or 5 or 6 or 7

9 limit 8 to randomized controlled trial

| Number of studies = 10 Root MSE = 1.037 | | | | | | |
| --- | --- | --- | --- | --- | --- | --- |
| Std_Eff | Coefficient | Std. err. | t | P> \|t\| | [95% conf. interval] | |
| Slope | -0.0131849 | 0.0156064 | -0.84 | 0.423 | -0.0491732 | 0.0228034 |
| bias | 2.447164 | 1.561196 | 1.57 | 0.156 | -1.152961 | 6.047289 |
| Test of H0: no small-study effects P = 0.156 | | | | | | |

Additional file 3 Assessment of the publication bias for serum creatinine

| Number of studies = 9 Root MSE = 1.25 | | | | | | |
| --- | --- | --- | --- | --- | --- | --- |
| Std_Eff | Coefficient | Std. err. | t | P> \|t\| | [95% conf. interval] | |
| Slope | -2.137922 | 2.952342 | -0.72 | 0.492 | -9.119103 | 4.843259 |
| bias | 0.6891386 | 2.474801 | 0.28 | 0.789 | -5.162836 | 6.541114 |
| Test of H0: no small-study effects P = 0.789 | | | | | | |

Additional file 4 Assessment of the publication bias for estimated glomerular filtration rate

Additional file 5 Assessment of the certainty of evidence of the GRADE approach

| **SGLT2 inhibitors compared to a placebo for renal outcomes in Japanese patients with type 2 diabetes** | | | | | | |
| --- | --- | --- | --- | --- | --- | --- |
| **Patient or population:** Japanese patient with type 2 diabetes **Settings:** Outpatients **Intervention:** SGLT2 inhibitors **Comparison:** Placebo | | | | | | |
| **Outcomes** | **Illustrative comparative risks* (95% CI)** | | **Relative effect (95% CI)** | **No of Participants (studies)** | **Quality of the evidence (GRADE)** | **Comments** |
|  | Assumed risk | Corresponding risk |  |  |  |  |
|  | **Placebo** | **SGLT2 inhibitors** |  |  |  |  |
| **SCr changes from baseline** Follow-up: 12-24 weeks |  | The mean SCr changes from baseline in the intervention groups was **0.01 higher** (0 to 0.02 higher) |  | 1955 (10 studies) | ⊕⊕⊝⊝ **low**^1,2^ | Compared with the placebo, there may be a slight difference in the SCr change. |
| **eGFR changes from baseline** Follow-up: 16-104 weeks |  | The mean eGFR changes from baseline in the intervention groups was **1.3 lower** (2.23 to 0.37 lower) |  | 1649 (9 studies) | ⊕⊕⊕⊝ **moderate**^3^ |  |
| *The basis for the **assumed risk** (e.g. the median control group risk across studies) is provided in footnotes. The **corresponding risk** (and its 95% confidence interval) is based on the assumed risk in the comparison group and the **relative effect** of the intervention (and its 95% CI).  **CI:** Confidence interval; SCr: Serum creatinine. | | | | | | |
| GRADE Working Group grades of evidence **High quality:** Further research is very unlikely to change our confidence in the estimate of effect.  **Moderate quality:** Further research is likely to have an important impact on our confidence in the estimate of effect and may change the estimate. **Low quality:** Further research is very likely to have an important impact on our confidence in the estimate of effect and is likely to change the estimate. **Very low quality:** We are very uncertain about the estimate. | | | | | | |
| ^1^ Two out of 10 studies were in terms of deviations from the intended interventions, while 5 were considered to have a high risk of bias in terms of missing outcome data. ^2^ The lower CI of 0 was considered to be a negligible effect as a patient-important outcome. ^3^ Two out of 9 studies were in terms of deviations from the intended interventions, while 4 were considered to have a high risk of bias in terms of missing outcome data. | | | | | | |
